# Evaluating the feasibility of conducting a trial using a patient decision aid in implantable cardioverter defibrillator candidates: a randomized controlled feasibility trial

**DOI:** 10.1186/s40814-017-0189-9

**Published:** 2017-11-21

**Authors:** Sandra L. Carroll, Dawn Stacey, Michael McGillion, Jeff S. Healey, Gary Foster, Sarah Hutchings, Heather M. Arthur, Gina Browne, Lehana Thabane

**Affiliations:** 1 Faculty of Health Sciences, School of Nursing, McMaster University, 1280 Main St. W, Hamilton, ON Canada; 2 Population Health Research Institute, Hamilton Health Sciences, Hamilton, ON Canada; 3 School of Nursing, University of Ottawa, Ottawa, Canada; 4 Ottawa Hospital Research Institute, Ottawa, Canada; 5 Department of Medicine, McMaster University, Hamilton, Canada; 6 Department of Health Research Methods, Evidence and Impact, McMaster University, Hamilton, ON Canada; 7 National University of Ireland, Galway, Ireland; 8 The Research Institute, St. Josephs’s Healthcare, Hamilton, Ontario Canada

**Keywords:** Implantable cardio-defibrillator, Decision aid, Shared decision-making, Knowledge translation

## Abstract

**Background:**

Patient decision aids (PtDA) support quality decision-making. The aim of this research was to evaluate the feasibility of conducting a randomized controlled trial delivering an implantable cardioverter defibrillator (ICD)-specific PtDA to new ICD candidates and examining preliminary estimates of differences in outcomes.

**Methods:**

Prior to recruitment, ICD candidacy was determined. Consented patients were randomized to (1) usual care or (2) PtDA intervention. Feasibility outcomes included referral and recruitment rates, successful PtDA delivery, and completion of measures. The PtDA intervention was administered prior to specialist consultation and baseline demographics, and measures of decision quality including decisional conflict (DCS), SURE test (Sure of myself, Understand information, Risk-benefit ratio, Encouragement), patient’s ICD specific values, ICD knowledge, and health-related quality of life were recorded. Post-consultation, participant’s DCS was repeated and decisions to proceed, decline, or defer ICD implantation were collected. Feasibility data was determined using descriptive statistics (continuous and categorical). Preliminary estimates of differences in outcomes were assessed using mean differences. Concordance between values and decision choice was assessed using logistic regression of the intervention group.

**Results:**

We identified 135 eligible patients. Eighty-two consented to the trial randomizing patients to usual care (*n* = 41) or PtDA intervention (*n* = 41). Feasibility outcome results were (1) referral rate at approximately 20/month, (2) recruitment rate 61%, and (3) successful delivery of PtDA and study management. Pre-consultation, PtDA patients scored lower on the DCS scale (mean, standard deviation [SD] 27.3 (18.4) compared to usual care, 49.4 (18.6); the between-group difference in means [95% confidence interval (CI)] was − 22.1[− 30.23, − 13.97]. A difference remained post-implantation 21.2 (11.7), PtDA intervention 29.9 (13.3), and usual care − 8.7 [− 14.61, − 2.86]. SURE test results supported DCS differences. The PtDA group scored higher on the ICD-related knowledge questions, with 47.50% scoring greater than 3/5 of the knowledge questions correct, compared to 23.09% receiving usual care. The mean [SD] number of correct knowledge responses out of 5 was 3.33(1.19) in the PtDA group and 2.62 (1.16) in usual care pre-implant. Concordance between values and decision choice found a strong association between predicted and actual ICD implant status in the intervention group.

**Conclusion:**

Our results suggest that a future definitive trial is feasible. The ICD-specific PtDA shows promise with respect to preliminary estimates of differences in outcomes.

**Trial registration:**

NCT01876173.

**Electronic supplementary material:**

The online version of this article (10.1186/s40814-017-0189-9) contains supplementary material, which is available to authorized users.

## Background

Sudden cardiac death (SCD), associated with lethal cardiac arrhythmias, is a leading cause of cardiovascular-related death in Canada [1], USA [2], and Europe [3, 4]. Implantable cardio-defibrillators (ICDs) can detect and successfully terminate these arrhythmias by delivering therapy in the form of an internal shock or anti-tachycardia pacing. Evidence generated from randomized controlled trials (RCTs) demonstrates mortality benefits for patients with ischemic and non-ischemic heart disease and congestive heart failure [5–10]. Current practice guidelines [11] recommend that patients who are at high risk (primary prevention) and patients who have experienced sustained ventricular arrhythmias (secondary prevention) are candidates for an ICD.

Delivery of ICD care can be complex, most often requiring access to an electrophysiologist for implantation and ongoing follow-up. For primary prevention patients in particular, the decision to receive this life-prolonging intervention may not be straightforward. Choosing to receive an ICD for primary prevention is considered an elective procedure, and thus, the option to accept or decline must be weighed alongside known benefits and risks. From the patient’s perspective, an ICD is a long-term intervention that involves a commitment to attend follow-up visits at a qualified center and to potentially undergo device replacements for batteries, lead revisions, or manufacturing advisories [12, 13]. Moreover, device-related complications (i.e., infection, lead dislodgement, inappropriate shocks) necessitate additional invasive procedures and driving privileges can be restricted (due to receipt of shock or anti-tachycardia pacing) [14–16]. However, the ICD has the potential for life-prolonging benefits when appropriate therapy is received. Given the need to weigh benefits and risk, the preferences of ICD candidates are essential in achieving higher quality decisions and decreasing unwarranted practice variation [17].

Rooted in the concept of patient-centered care, shared decision-making encourages patients to be active participants when making healthcare-related treatment decisions. To achieve quality decision-making (i.e., informed, value-based), a decision support process which presents patients with balanced evidence-based facts, associated risks and benefits, and an assessment of patient’s values and preferences is imperative [18]. Patient decision aids (PtDA) have been developed to facilitate quality decision-making, ensuring congruency between patient values and preferences and the health care interventions they choose. A recent Cochrane Review of PtDA demonstrated evidence that PtDA improved patients’ knowledge, accurate risk perception, and reduced decisional conflict [19]; all of which contribute to what is considered a quality decision.

Our previous research with ICD candidates suggested that very few patients engaged actively in their ICD decision-making process, expressing uncertainty regarding ICD options [20, 21]. Given the complexities associated with the ICD as an intervention and identified patient needs, we endeavored to support decision-making by developing an ICD-specific PtDA for patients offered their first device. The primary objective of this study was to (1) test the feasibility of conducting a RCT in ICD candidates considering the device. The secondary objectives were to (2) evaluate the preliminary effects of the ICD-specific PtDA and (3) determine if differences in preliminary effects (decision quality) are present post-ICD implantation.

## Methods

### Pilot trial design

We undertook a two-arm parallel-group pilot RCT of a newly developed PtDA [22]. Our detailed methods and protocol have been reported previously [23]. Guided by the International Patient Decision Aid Standard (IPDAS) in terms of content, development process, and effectiveness [24, 25] and the Ottawa Decision Support Framework [26], we developed an ICD-specific PtDA. Following informed consent procedures and study introduction, patients were randomized to receive PtDA intervention or usual care using a 1:1 allocation ratio. Randomization took place prior to electrophysiology specialist consultations using a centralized Internet randomization service (https://www.randomize.net); permuted blocks of 4 and 6 were used to balance treatment arms. The use of https://www.randomize.net ensured that the allocation sequence was concealed from the research assistant. Due to the nature of the intervention, patients and the research assistant collecting data were not blinded to study group assignment. The data analyst was blinded to group assignment.

### Setting and participants

The study took place at a large tertiary care center in Hamilton, Ontario, Canada. The center, Hamilton Health Sciences (HHS), serves approximately 2.3 million residents and is a regional referral center for specialized arrhythmia and cardiovascular services.

Eligible patients included those referred for a primary prevention ICD. At the study site, the majority of patients are referred to the arrhythmia service by cardiologists and heart failure specialists in the region. All referrals are triaged by a registered nurse (based on guidelines) and procedure wait times tracked by the Cardiac Care Network, a provincial support system serving Ontario. Following referral, consultations are booked with an electrophysiologist and the patient. Exclusion criteria were (1) an inability to understand the PtDA because of a language barrier or visual impairment and (2) referral for cardiac resynchronization devices (CRT). At the time of study conception, specialist consultation wait times were 4–6 weeks following initial referral. With added efficiencies to manage patient wait times, our center’s wait times for specialist consult were reduced to 2–3 weeks. To adapt to this change in process, we amended our study to accommodate the shorter wait times—eliminating mail invitations for the study and inviting patients to meet with the nurse research assistant (RA) at HHS prior to specialist consultation (at least 60 min). The study was introduced to patients by members of the healthcare team (i.e., triage nurse or physician) when the referral was initiated and consultations arranged. Patients randomized to the intervention group (PtDA) received the PtDA to review and complete *prior* to their specialist consultation directly with a trained health professional research assistant. The RA assured that participants received the PtDA in advance, were provided with adequate time to review the PtDA in full, and answer questions about the PtDA process. Patients were encouraged to record and communicate specific ICD questions to bring to their specialist consult which followed.

### PtDA intervention

To summarize briefly, the PtDA was devised and tested in three steps. Step 1 was the development of the PtDA, using an inter-disciplinary panel (including patients) where the PtDA content was assessed, drafted, and revised. The PtDA has five structured steps to guide patients through options as follows: (1) Be clear about the decision (i.e., makes explicit decision); (2) think about the benefits and risks (options including probabilities of benefits and risks); (3) what matters most to patients (values clarification); (4) what role is preferred by patients in decision-making; and (5) what else do you need? (knowledge questions). The knowledge and value items are included (see Additional file 1).

To calculate risk and benefit probability estimates for inclusion in the PtDA, evidence from RCTs that compared ICDs to conventional therapy in patients receiving ICD prophylaxis without cardiac resynchronization (CRT) was pooled. Patient outcomes were extracted for (1) all-cause mortality, (2) cardiac mortality, (3) shocks (appropriate/ inappropriate), (4) infection, and (5) complications, where reported. We employed an analytic modeling technique, as follows: Probabilities and associated 95% confidence intervals were calculated from individual studies using the exact binomial method. Overall probabilities were calculated by pooling the probabilities from available RCTs using random-effects meta-analysis based on the inverse variance weighted approach [27]. Selected benefit and risk probabilities [28] were then incorporated into the PtDA using text and pictograms. The inter-disciplinary development panel collaborated to provide input on the knowledge, values, background content, and format (e.g., font size, readability, style) of the PtDA. We employed a two-round Delphi process with the panel, health professionals with ICD expertise, and patients (with ICDs) to reach consensus on final PtDA content [28]. Step 2 included preliminary acceptability testing in patients and families with experiential knowledge of ICDs [29]. Feedback was incorporated into the PtDA. The final result was an 11-page interactive PtDA booklet that presents ICD candidates and their families with explicit evidence-based information and probabilities about the benefits and risks associated with an ICD using lay terminology.

### Usual care

Patients randomized to usual care did not receive additional information from the study team prior to specialist consult. Point of delivery of general education material to patients varies across arrhythmia centers but is generally delivered *after* consultation once the decision to accept the ICD is established. Manufacturer-specific booklets are also provided after implantation but prior to discharge. Preoperative ICD specific material is disseminated after patients are consented for the procedure.

### Outcomes

The primary outcome for this trial was feasibility of conducting the RCT measured using (1) rate of recruitment, (2) successful delivery and completion of the PtDA (including randomization technique), and (3) completion of measures. The secondary outcomes were to evaluate the preliminary estimates of differences in outcomes between as measured by knowledge scores, decisional conflict scores, and the SURE (Sure of myself, Understand information, Risk-benefit ratio, Encouragement) test for screening for decisional conflict. Both PtDA intervention and usual care groups completed study measures including the Decisional Conflict Scale (pre- and post-specialist consultation), knowledge questions (pre-consultation), and the Preparation for Decision-Making Scale [30, 31] preparedness questionnaire (PtDA intervention, pre-consultation only).

### Baseline measurements and patient demographics

When capturing basic demographic characteristics of our patient population (age, sex, education, employment status, and ethnicity), we also measured baseline psychosocial status, including health-related quality of life (HRQOL) and depressive symptoms. We employed the Medical Outcomes Trust Short Form (SF-36v2), a generic scale widely used in healthcare to measure HRQOL. The SF-36v2 has two component summary measures, physical component summary (PCS) and mental component summary (MCS), which shed light on the groups’ physical and mental health status, respectively. The SF-36V2 uses a norm-based scoring method, wherein raw scale values from 0 to 100 are transformed. The transformation results in a mean score of 50 and SD of 10. Normal PCS and MCS scores range from 45 to 55 [32, 33]. To capture depressive symptoms, we used the 20-item Center for Epidemiological Studies Depression Scale (CES-D). Patients received a summed score between 0 and 60, where a score greater than 16 is indicative of mild to moderate depressive symptomatology [34, 35]. Both instruments have demonstrated validity and reliability across different clinical settings [36–39].

### Outcome evaluation measures

#### Knowledge and values

In order to evaluate decision quality of patient participants, we employed two measures of decision quality. Patients’ ICD-related knowledge was assessed using five knowledge-based questions developed by the inter-disciplinary panel (see Additional file 1). For example, a true, false, or unsure item was “In the future, I can choose to ask my doctor to turn off (deactivate) ICD therapy.” In addition, six value items were developed and measured in the intervention group (due to ethics concerns). Examples of items included “How important is it to you to lower your chances of sudden cardiac death?” and “How important is it to you to avoid complications from an ICD?” (see Additional file 1). Value and knowledge items were informed by the Delphi, inter-disciplinary panel, and previous research [20, 28, 29]. The six value items were scored from 1 to 5: 1 = Not important to 5 = Very important.

### Decisional Conflict Scale

To measure the patients’ perception of their difficulty in making decisions, level of uncertainty, personal values, and perceived support in making their decision, we used the Decisional Conflict Scale (DCS). The DCS is a validated tool made up of 16 items, which provide an overall DCS score and five subscale scores (Informed, Values Clarity, Support, Uncertainty and Effective Decision). Higher DCS scores are indicative of decisional conflict which can lead to delayed decision-making, vacillation between treatment choices, and decisional regret including signs of distress [40]. DCS scores range from 0 to 100; scores > 37.5 are associated with decisional delay and feelings of uncertainty [40]. The DCS has established reliability and validity [19, 41, 42].

### The Sure of myself, Understand information, Risk benefit ratio, Encouragement Test (SURE Test)

The SURE test is a 4-item screening test developed to quickly assess decisional conflict (certainty, knowledge, values, support) in the consult setting [43]. The SURE test has been validated in adult patients [44] but not ICD patients specifically. Following completion of the DCS questionnaire, we asked patients to also complete the SURE test. Each item on the SURE test has two response categories “yes” or “no.” If patients answer “no” to any of the four SURE items, it suggests the presence of decisional conflict. Healthcare professionals are able to identify patients’ specific area of conflict based on items selected as “no” during consultation and undertake further discussions. The standardized Cronbach’s alpha in our sample was 0.75. This is an acceptable measure of internal consistency suggesting that the items are reasonably related as a group.

### Measures post-consultation—Preparation for Decision-Making Scale

In the PtDA group, patients’ perception of usefulness of the PtDA in preparation for consultation with a specialist was assessed using the Preparation for Decision-Making Scale (PDMS) at the 2-week follow-up. The PDMS contains 10 items. Total scores range from 0 to 100, where higher scores are indicative of a higher level of perceived preparation for making a treatment decision. The PDMS has demonstrated good reliability [31].

### Three-month ICD implantation status

In addition, patient’s ICD 3-month ICD implantation status (ICD implanted, declined, or deferred) was obtained from the medical record.

#### Sample size

The study’s primary outcome was to assess the feasibility of successfully conducting a larger trial. The sample size for this study was determined based on feasibility considerations [45, 46]. Our recruitment target was set at 80 patients: 40 patients per arm, large enough to provide useful estimates of data [47], and feasibility, based on rate of standard ICD referrals from the previous year (approximately 300) and number of patients that would have fit our eligibility criteria in the past. Furthermore, our sample from Hamilton Health Sciences was representative of the target study population (elective primary prevention ICD candidates).

#### Data analysis

To maximize data quality, standardized data collection forms were developed. Data were entered electronically following facsimile transmission using Teleform (Cardiff Teleform). Study feasibility data, including referral rates and recruitment rates, were determined using descriptive statistics. Continuous variables were summarized using descriptive statistics and measures of central tendency (means, standard deviations). Categorical variables were summarized using percentages (counts). Exploration of preliminary estimates of differences in outcomes between groups, including decisional conflict, was conducted using mean difference and differences between group means (pre-post consult). Knowledge scores were evaluated by comparing the mean number of correct responses between groups and by comparing the proportion of patients with greater than three correct responses. As this is a pilot trial, these preliminary estimates of differences in outcomes and associated confidence intervals will be useful in the design of a definitive trial in informing sample size calculation.

To determine if the predicted status of an ICD (based on the set of 6 value items) is associated with ICD implantation status at 3 months post-consultation, concordance was assessed using logistic regression (restricted to the intervention group). The actual ICD status is determined at 3 months post-consultation and could be either (1) ICD implanted, (2) ICD declined, or deferred. The six value items were scored from 1 to 5: 1 = Not important to 5 = Very important. Value items 5 and 6 where then reverse-scored. These six value items were then re-scored for the purposes of the concordance analysis such that a value item was deemed important if the value score was 4 or 5 and not important if the score was 1, 2, or 3.

The predicted status of an ICD was computed using the results of a logistic regression analysis where the actual ICD implantation status was the outcome and the six re-scored value items the predictors. The model yields a predicted probability of receiving and ICD for each patient. If patient’s predicted probability is less than or equal to 0.5, that patient’s predicted ICD status would be “No ICD.” Likewise, a predicted probability of greater than 0.5 would result in a predicted ICD status of “ICD implanted.”

The SURE test scores were evaluated using differences in percentages (PtDA–usual care). The results are reported as preliminary estimates of differences in outcomes and corresponding 95% confidence intervals (CI). All analyses were conducted using SAS 9.3 (Cary, NC, USA).

## Results

### Characteristics of the sample

The majority of patients were male (73.2%), aged 67 years, Caucasian (94%), living with someone (82.0%), and retired (60.0%) (Table 1). Most had hypertension (67.0%) and underlying ischemic heart disease (58.4%). Almost half had a history of myocardial infarction (47.6%), and 34.2% reported a history of congestive heart failure. Overall, the characteristics of the sample were balanced between groups with the exception of diabetes which was higher in the intervention group. HRQL and CES-D data revealed patients in both groups reported similar scores for depressive symptoms, lower SF-36 physical component summary scores, and population norm mental component summary scores [32, 33]. In terms of medical management, the sample (usual care vs. PtDA intervention) at the time of enrolment was balanced.

**Table 1:** Baseline patient characteristics and history (*N* = 82)

Characteristic	Usual care *n* = 41	PtDA intervention *n* = 41
Age: mean (SD)	67.2 (12.6)	66.3 (9.4)
Male	30 (73.2)	30 (73.2)
Living with someone	29 (70.3)	38 (92.7)
Education		
High school	20 (52.6)	21 (55.3)
College/trade	13 (34.2)	14 (36.8)
University/graduate school	5 (13.1)	3 (7.9)
Current employment status		
Full-time/part-time	4 (9.8)	9 (22)
Retired	27 (65.9)	22 (53.7)
Disability	10 (24.4)	10 (24.4)
Ethnicity		
Caucasian	36 (87.8)	41 (100)
Other	4 (9.8)	0 (0.0)
CES-D: mean (SD)	16.6 (4.5)	14.5 (6.2)
SF36-v2: mean (SD)		
MCS	48.5 (12.6)	51.4 (13.2)
PCS	37.1 (7.7)	36.7 (9.3)
Cardiovascular history		
Previous MI	18 (43.9)	21 (51.2)
Previous CABG	8 (19.5)	7 (17.1)
Previous stroke or TIA	6 (14.6)	4 (9.8)
Hypertension	13 (31.7)	15 (36.6)
Ischemic CAD	26 (63.4)	26 (63.4)
Non-ischemic	12 (29.3)	5 (12.2)
CHF history	22 (53.7)	29 (70.7)
Atrial fibrillation	16 (39.0)	7 (17.1)
Ejection fraction mean (SD) comorbidities	26.1 (4.4)	27.8 (6.0)
Diabetes	12 (29.3)	24 (58.9)
COPD	7 (17.1)	6 (14.6)
Recorded NYHA (at referral)		
Class I	4 (9.8)	4 (9.8)
Class II	16 (39.0)	16 (39.0)
Class III	11 (26.8)	8 (19.5)
Undocumented	10 (24.4)	13 (31.7)
Medication		
ACE inhibitor	28 (68.3)	26 (63.4)
β-blocker	35 (85.4)	32 (78.1)
Ca^2+^ channel blocker	5 (12.2)	6 (14.6)
Diuretic	28 (68.3)	24 (58.5)
Insulin/diabetic medication	11 (26.8)	22 (53.7)
Lipid lowering agent	28 (68.3)	25 (61.0)
Anti-depressant	6 (14.6)	2 (4.9)

CES-D score greater than 16 indicates mild to moderate depressive symptoms. Normal MCS and PCS scores fall from 45 to 55. Values are expressed as *n* (%) unless otherwise indicated

*CES-D* Center for Epidemiological Studies Depression Scale, *MCS* Mental Component Scale, *PCS* Physical Component Scale, *SD* standard deviation, *MI* myocardial infarction, *CABG* coronary artery bypass graft, *TIA* transient ischemic attack, *CAD* coronary artery disease, *CHF* congestive heart failure, *COPD* chronic obstructive pulmonary disease, *NYHA* New York Heart Association, *ACE* angiotensin-converting enzyme

**Fig. 1:**
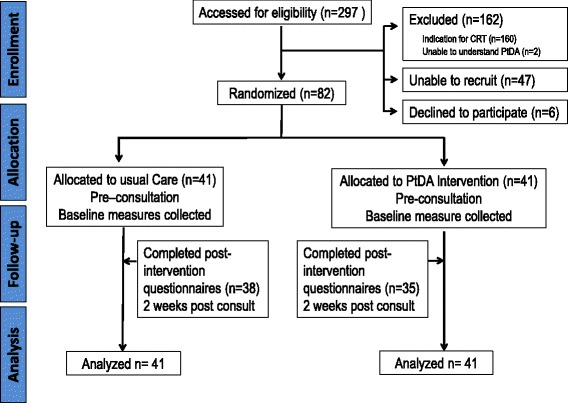
Consort flow diagram. Outlining patient recruitment, enrollment, allocation, and analyses

Figure 1 provides the CONSORT [48] diagram for the study. The CONSORT extension for pilot and feasibility trials is included (see Additional file 2). As stated in our methods, the original study protocol [23] aimed to invite patients by mail. Our reduced wait times limited the ability of patients to return invitations and PtDAs by mail prior to specialist consult, and thus, the study protocol was amended prior to commencing to allow us to invite candidates when they attended the outpatient clinic for consultation following study introduction.

### Feasibility outcomes

Over a 14-month period (2013–2014), 297 patients were screened for eligibility (average referral rate = 20 patients/month). A total of 162 patients were deemed ineligible; the primary reason for ineligibility was an indication for cardiac resynchronization therapy (CRT) device (53.9%). Of the 135 remaining patients, a recruitment rate of 61% was achieved. The remaining 35% of eligible patients were not able to be approached by our RA primarily for logistical reasons including patient’s clinic time or schedule changes and availability of research staff to attend clinic. Eighty-two patients consented to participate and were successfully randomized to usual care (*n* = 41) or PtDA intervention (*n* = 41). All patients who attended the clinic and received the PtDA were able to complete the PtDA with the RA. The percentage of missing data was low (< 5%) and mainly restricted to the post-implantation follow-up surveys (where DCS follow-up and PDMS were not returned by mail and patients did not respond to requests). Feasibility outcomes and a priori criteria for success are summarized in Table 2.

**Table 2:** Feasibility results for the PtDA trial

Measure	Observed	Target: a priori criteria for success	Description
Referral rate Primary prevention ICD (14 months)	21 per month	Consistent with referral patterns previously for these candidates	Patients referred for all devices, then triaged based on guidelines
Sample size (*N*)	82	80	Accomplished target
Recruitment rate	61%	80%	Barriers to access and resources influenced rate of recruitment
Delivery of PtDA to intervention group	100%	80%	All patients randomized to PtDA intervention received it
Completion of PtDA to intervention group	100%	80%	All patients met with nurse research assistant to complete PtDA steps
Completion of quality measures	< 10% items missing	80%	Items from HRQL measures and post-implant

### Secondary outcomes

#### Knowledge and values

The PtDA intervention group participants were more knowledgeable after exposure to the PtDA pre-specialist consultation. The number (%) of participants in the PtDA group scoring greater than 3/5 of the knowledge questions correct 19 (47.5) compared to 9 (23.1) of patients receiving usual care. The mean (SD) number of correct responses for the intervention and usual care groups was 3.33 out of 5 (1.19) and 2.62 (1.16), respectively.

Values in the PtDA intervention group measured after exposure to the PtDA and pre-consultation revealed that the majority of patients (> 65%) scored items as “high importance” (5/5) across 5 of the 6 items with the exception of “How important is it for you to avoid shocks from an ICD?” at 58% (see Additional file 1). The results for concordance between patient’s values and decision choice confirm that there is a strong association between predicted and actual ICD status (*p* = 0.017) when all six values are used a predictors in the model. Patients who received an ICD were more consistent with their values (20/22 or 90.9%) than patients who did not receive an ICD (7/15 or 46.7%). The percentage of all patients in the intervention group for whom the predicted and actual ICD status was the same is 27/37 or 73%.

### Decisional conflict

Patients receiving the PtDA intervention had lower decisional conflict scores after exposure to the PtDA pre-specialist consult, with a total mean score (SD) of 27.3 (18.4) compared to usual care 49.4 (18.6) (Table 3). The mean decisional conflict total score remained lower in the PtDA intervention group 21.2 (11.7) compared to the usual care group 29.9 (13.3) post-implantation (Table 3). The post-implantation difference between usual care and PtDA intervention group DCS means [95%CI] was − 8.7 [− 14.61, − 2.86]. All five baseline DCS subscale mean scores (informed, values clarity, support, uncertainty, and effect decision) were lower in the PtDA intervention group and remained lower in the intervention group post-ICD implantation.

**Table 3:** Decisional conflict scores post-PtDA prior to specialist consultation and post-implantation

Characteristic	Usual care *N* = 41 Mean (SD)	PTDA intervention *N* = 41 Mean (SD)	Difference in group means PTDA—UC [95% CI]
DCS total score prior to consultation	49.4 (18.6)	27.3 (18.4)	− 22.1 [− 30.23, − 13.97]
DCS subscale			
Informed	59.8 (22.0)	29.1 (21.5)	
Values clarity	56.9 (23.0)	25.8 (17.5)	
Support	37.2 (18.3)	23.6 (18.0)	
Uncertainty	54.9 (25.6)	31.1 (24.6)	
Effect decision	41.2 (20.4)	27.1 (20.2)	
Characteristic	Usual care *n* = 38 Mean (SD)	PtDA intervention *n* = 35 Mean (SD)	Difference in group means PtDA—UC [95% CI]
DCS total score post-implantation	29.9 (13.3)	21.2 (11.7)	− 8.7 [− 14.61, − 2.86]
DCS subscale			
Informed	28.5 (12.7)	21. 2 (13.5)	
Values clarity	31.4 (17.9)	22.6 (14.4)	
Support	26.1 (12.7)	17.9 (13.3)	
Uncertainty	34.4 (17.5)	25.2 (17.8)	
Effect decision	29.3 (15.2)	19.5 (12.5)	

### The SURE test

A higher percentage of patients in the PtDA intervention group answered “yes” to the knowledge, values, and certainty items (Table 4). A higher percentage *n* (%) of participants in the intervention group responded “yes” to the support item 34 (82.9) vs. usual care 28 (68.3). Table 4 shows the percentages  for each  group using the SURE test. These findings corroborate the full instrument DCS scores using the 16-item scale. The PtDA intervention group reported lower decisional conflict on both measures. For the four SURE items, standardized Cronbach’s alpha was 0.75. This is an acceptable measure of internal consistency suggesting that the items are reasonably related as a group. Overall, 19 patients answered “yes” to all four items on the SURE test, 16 of these were in the PtDA group.

**Table 4:** Group percentages using the SURE test

Characteristic	Usual care *n* = 41 *n* (%)	PtDA intervention *n* = 41 *n* (%)
Certainty		
Do you feel sure about the best choice for you?	10 (24.4)	21 (51.2)
Knowledge		
Do you know the benefits and risks of each option?	4 (9.8)	26 (63.4)
Values		
Are you clear about which benefits and risks matter most to you?	9 (22.0)	32 (78.1)
Support		
Do you have enough support and advice to make a choice?	28 (68.3)	34 (82.9)

### Preparation for decision-making

In the intervention group, patients’ mean (SD) preparedness score was 67.3 out of 100 (23.5) when asked to recall their preparedness to make their decision at the post-ICD implantation follow-up.

### Three-month ICD implantation status

We assessed ICD implantation status 3 months post-consultation finding 24 out of 41patients or 58.6% [42.8, 74.3] who received usual care and 24/41 patients or 58.6% [42.8, 74.3] in the PtDA intervention group who had an ICD implanted. Five patients or 12.2% [1.7, 22.7] in the usual care group declined an ICD and 2 or 4.9% [0.0, 11.8] declined an ICD in the PtDA intervention group. More patients made the choice to defer their ICD rather than decline, with 11 or 26.8% [12.7, 41.0] in usual care and 14 or 34.2% [19.0, 49.3] in the PtDA group, respectively. Overall, no difference in 3-month ICD implantation status between usual care and PtDA intervention was found.

## Discussion

To our knowledge, this is the first feasibility RCT to implement a PtDA in primary prevention ICD patients. Our findings suggest that a definitive large-scale RCT is feasible and that our ICD-specific PtDA demonstrated positive change in outcomes.

### Feasibility

The first aim of this study was to assess the feasibility of conducting an RCT. Our criteria for feasibility aimed for an 80% recruitment rate of eligible patients, an 80% delivery and completion rate of the PtDA, and an 80% completion rate of all questionnaires (DCS, PMDS, etc.). Only six of the 135 eligible patients (4.4%) declined to participate in the study. Several of the non-recruited candidates had agreed to meet with the RA for the study but then experienced schedule changes. Thus, our recruitment rate of 61% does not necessarily depict unwillingness on the part of patients but our inability to access them. For example, one logistical barrier persisted when patients entered consultation rooms before meeting with the nurse RA to consent, undergo randomization, and receive the PtDA (if randomized to the intervention group). This recurred when physicians saw patients earlier than scheduled because they had arrived early to meet the RA. Clinic settings are not always ideal but often the only option available to researchers when conducting clinical research. Interruptions that impact existing clinic/care processes may not be welcomed by staff; thus, there is a need to strike a balance that maximizes quality data collection and minimizes disruption to processes in clinic settings. Identification of logistical barriers in this pilot trial will inform a future trial and the need to involve clinic staff in the design if shared decision-making is a goal in health organizations. However, once participants were enrolled, targets for PtDA delivery and completion rates were achieved using our amended protocol. Furthermore, utilizing https://www.randomize.net via smartphone allowed for rapid randomization, eliminating the need to access a computer terminal. We are currently developing an electronic version of this PtDA to offer web-based access.

The 16-item DCS questionnaire required more time than expected to complete, which with the already large demand for time in the ICD clinic setting may therefore not be feasible in everyday practice. For this reason, we employed the 4-item SURE test, a concise measure to screen for decisional conflict. In our sample, results of the SURE test corroborated the DCS questionnaire results; the PtDA appeared to have increased patient knowledge, certainty, and values consistently, but not perceived support.

### Preliminary estimates of differences in outcomes between groups

The second aim was to estimate the preliminary estimates of differences in outcomes between groups using an ICD-specific PtDA. Administration of our PtDA found 24.4% of the PtDA group scored greater than 3/5 of the knowledge questions correct and reported lower decisional conflict where the mean group difference was − 22.1[− 30.23, − 13.97; PtDA–UC]. These findings are corroborated by published PtDA research across other patient populations including ischemic heart disease [49], atrial fibrillation [50, 51], and candidates for elective open-heart surgery [52]. A recent meta-analysis of 115 PtDA trials, 42 of which recorded knowledge scores in varying disease populations, found mean knowledge scores were 13.3% higher, on average, in the PtDA intervention groups compared to controls [19]. Patients require knowledge to make educated and informed decisions regarding their health [53]. Today, many patients turn to the Internet for health information and the volume of information can be overwhelming and of variable quality. Patients arrive for specialist consultations with knowledge gleaned from a variety of sources—including other ICD patients [54]. Furthermore, many do not have the requisite skills to appraise quality of information sources from a consumer perspective. PtDA’s provide patients with valuable, quality information in a sufficient amount to support decision-making.

Lower decisional conflict scores following administration of a disease-specific PtDA have been reported in other populations of patients considering elective surgery, such as mastectomy [55], and prostatectomy [56]. In accordance with the previous PtDA research, we found ICD candidates who received the PtDA intervention experienced less decisional conflict. Prior to the initial specialist consult, the usual care group’s total DCS scores were extremely high, well over 37.5, the value suggested to be likely to increase the risk of decisional delay and feelings of uncertainty [40]. The PtDA intervention group total DCS score was lower post-PtDA exposure, yet these patients still experienced some degree of decisional conflict. The total DCS score was close to the 25 cut-off mark, which is associated with higher likelihood for making the decision [40]. Arguably, some decisional conflict is expected when making life-prolonging healthcare decisions involving surgery such as implantation of an ICD, regardless of whether patients receive additional decision support. Compared to usual care, the PtDA intervention group reported less decisional conflict in four of the five DCS subscales (informed, values clarity, uncertainty, and effect decision) with the exception of support. We postulate the PtDA intervention encouraged patients to consider their support (i.e., support to make a choice, choosing without pressure, and enough advice), but ultimately, did not alter whether or not the support in fact exists, and thus, their perception of support did not differ.

The strong association between value items and ICD implantation is encouraging and warrants inclusion of a values clarification exercise in a future definitive trial. Concordance of the six value items in the PtDA group revealed the item with the strongest contribution was “How important is it to you to lower your chances of a sudden cardiac death?”. This is not surprising in our quest to assess patient’s values, avoiding death prevailed in our sample. Clarification of patient values and preferences remains a challenge to elicit [57]. This can also be seen where our value item which addressed preference for a “natural death” did not reveal an association. Furthermore, values clarification was limited to the intervention group and does not allow for comparison with usual care. In practice, these value items are generally not addressed at the time of specialist consult.

The third study aim was to examine the post-implantation decisional conflict. In general, post-implantation decisional conflict dissipated, with both groups reporting lower DCS scores. The PtDA intervention group continued to report lower DCS scores relative to usual care. Although the differences post-implantation were not nearly as large, they could be clinically meaningful and revealed in a definitive effectiveness trial. The total mean DCS score for the usual care group remained greater than 25 post-consultations, suggesting a decision is unlikely. In contrast, the mean total DCS score in the PtDA intervention group remained lower than 25 post-implantation, suggesting maintenance of a difference in the PtDA intervention minimizing decisional conflict. Both groups demonstrated improvements in DCS following conversations with a specialist and arrhythmia team when we contacted patients post-consult. A larger sample and trial would allow for definitive answers.

### Limitations

There are several limitations to consider for this study. First, the population from which we drew our sample was limited to patients attending a specialized center. Hence, our results are not generalizable to general hospital settings which care for cardiovascular patients who could benefit from access to decision support prior to referral to specialized electrophysiology care.

Second, we do not know the outcome of the patients who at 3 months deferred their decision to go forward with an ICD. The reasons for deferral can vary where patients may have additional health conditions which are pressing, or patient preferences for timing to undergo a surgical procedure. Given the lower SF-36 PCS scores and presence of co-morbidities in our sample, this patient population is burdened with other health conditions. Our previous decision-making research in this population found that when patients deferred, often a new health event (related or not related) triggered the decision to receive the ICD [20].

Finally, the timing of PtDA delivery to ICD candidates in this feasibility trial was based in part on our previous findings where new ICD candidates suggested they had not engaged in the decision-making process prior to or during specialist consultation [19]. Moving forward, the inclusion of a sub-analysis in a future trial that assesses the timing effect of PtDA delivery could advance this line of inquiry and perhaps offer broader applicability to ICD centers where care pathways differ for patients entering the system.

## Conclusion

Application of a PtDA in eligible ICD candidates was feasible, and preliminary estimates found improved patient knowledge and lower decisional conflict. These findings have important implications for clinical practice and future research. Conducting a full-scale definitive RCT is an important next step to confirm our preliminary estimates in a larger sample of ICD candidates. Our results suggest that a PtDA contributed to quality decision-making with improved knowledge compared to usual care and chosen options that were congruent with patient values in the context of ICD implantation. PtDAs and decision coaching are effective interventions to engage patients sharing difficult decision-making together with their interprofessional teams. Determining successful approaches to mobilize these interventions into arrhythmia care could lead to improved patient reported outcomes.

## Additional files

Additional file 1: Knowledge and value items included in the Patient Decision Aid. (PDF 76 kb)

Additional file 2: CONSORT 2010 checklist of information to include when reporting a randomised trial*. (DOC 218 kb)

## Abbreviations

ACE: Angiotensin-converting enzyme
CABG: Coronary artery bypass graft
CAD: Coronary artery disease
CES-D: Center for Epidemiological Studies Depression Scale
CHF: Congestive heart failure
CIHR: Canadian Institutes of Health Research
COPD: Chronic obstructive pulmonary disease
CRT: Cardiac resynchronization therapy
DCS: Decisional Conflict Scale
HRQOL: Health-related quality of life
IASP: International Patient Decision Aid Standard
ICD: Implantable cardio-defibrillators
MCS: Mental component summary
MI: Myocardial infarction
NYHA: New York Heart Association
PCS: Physical component summary
PDMS: Preparation for Decision-Making Scale
PtDA: Patient decision aid
RA: Research assistant
RCT: Randomized controlled trial
SCD: Sudden cardiac death
SF36v2: Medical Outcomes Trust Short Form
SURE: Sure of myself, Understand information, Risk benefit ration, Encouragement
TIA: Transient ischemic attack

## References

[1] Tang AS, Ross H, Simpson CS, Mitchell LB, Dorian P, Goeree R, Hoffmaster B, Arnold M, Talajic M, on behalf of the Canadian Heart Rhythm S and the Canadian Cardiovascular S (2005). Canadian Cardiovascular Society/Canadian Heart Rhythm Society position paper on implantable cardioverter defibrillator use in Canada. Can J Cardiol.

[2] Lloyd-Jones D, Adams RJ, Brown TM, Carnethon M, Dai S, De SG, Ferguson TB, Ford E, Furie K, Gillespie C, Go A, Greenlund K, Haase N, Hailpern S, Ho PM, Howard V, Kissela B, Kittner S, Lackland D, Lisabeth L, Marelli A, McDermott MM, Meigs J, Mozaffarian D, Mussolino M, Nichol G, Roger VL, Rosamond W, Sacco R, Sorlie P, Roger VL, Thom T, Wasserthiel-Smoller S, Wong ND, Wylie-Rosett J, American Heart Association Statistics C and Stroke Statistics S (2010). Heart disease and stroke statistics—2010 update: a report from the American Heart Association. [Erratum appears in Circulation. 2010 Mar 30;121(12):e260 Note: Stafford, Randall [corrected to Roger, Veronique L]]. Circulation.

[3] Byrne R, Constant O, Smyth Y, Callagy G, Nash P, Daly K, Crowley J (2008). Multiple source surveillance incidence and aetiology of out-of-hospital sudden cardiac death in a rural population in the West of Ireland. Eur Heart J.

[4] Priori SG, Aliot E, Blomstrom-Lundqvist C, Bossaert L, Breithardt G, Brugada P, Camm AJ, Cappato R, Cobbe SM, Di Mario C, Maron BJ, McKenna WJ, Pedersen AK, Ravens U, Schwartz PJ, Trusz-Gluza M, Vardas P, Wellens HJJ, Zipes DP (2001). Task Force on Sudden Cardiac Death of the European Society of Cardiology. Eur Heart J.

[5] Connolly SJ, Hallstrom AP, Cappato R, Schron EB, Kuck KH, Zipes DP, Greene HL, Boczor S, Domanski M, Follmann D, Gent M, Roberts RS (2000). Meta-analysis of the implantable cardioverter defibrillator secondary prevention trials. AVID, CASH and CIDS studies. Antiarrhythmics vs Implantable Defibrillator study. Cardiac Arrest Study Hamburg. Canadian Implantable Defibrillator Study. Eur Heart J.

[6] Moss AJ, Zareba W, Hall WJ, Klein H, Wilber DJ, Cannom DS, Daubert JP, Higgins SL, Brown MW, Andrews ML, Multicenter Automatic Defibrillator Implantation Trial III (2002). Prophylactic implantation of a defibrillator in patients with myocardial infarction and reduced ejection fraction. N Engl J Med.

[7] Bardy GH, Lee KL, Mark DB, Poole JE, Packer DL, Boineau R, Domanski M, Troutman C, Anderson J, Johnson G, McNulty SE, Clapp-Channing N, vidson-Ray LD, Fraulo ES, Fishbein DP, Luceri RM, Ip JH, the Sudden Cardiac Death in Heart Failure Trial I (2005). Amiodarone or an implantable cardioverter-defibrillator for congestive heart failure. N Engl J Med.

[8] Kadish A, Dyer A, Daubert JP, Quigg R, Estes NA, Anderson KP, Calkins H, Hoch D, Goldberger J, Shalaby A, Sanders WE, Schaechter A, Levine JH, Defibrillators in Non-Ischemic Cardiomyopathy Treatment Evaluation I (2004). Prophylactic defibrillator implantation in patients with nonischemic dilated cardiomyopathy. N Engl J Med.

[9] Bristow MR, Saxon LA, Boehmer J, Krueger S, Kass DA, De MT, Carson P, DiCarlo L, DeMets D, White BG, DeVries DW, Feldman AM, Comparison of Medical Therapy PaDiHFI (2004). Cardiac-resynchronization therapy with or without an implantable defibrillator in advanced chronic heart failure. N Engl J Med.

[10] Moss AJ, Hall WJ, Cannom DS, Daubert JP, Higgins SL, Klein H, Levine JH, Saksena S, Waldo AL, Wilber D, Brown MW, Heo M (1996). Improved survival with an implanted defibrillator in patients with coronary disease at high risk for ventricular arrhythmia. Multicenter automatic defibrillator implantation trial investigators. N Engl J Med.

[11] Epstein AE, DiMarco JP, Ellenbogen KA, Estes NA, Freedman RA, Gettes LS, Gillinov AM, Gregoratos G, Hammill SC, Hayes DL, Hlatky MA, Newby LK, Page RL, Schoenfeld MH, Silka MJ, Stevenson LW, Sweeney MO, Smith SC, Jacobs AK, Adams CD, Anderson JL, Buller CE, Creager MA, Ettinger SM, Faxon DP, Halperin JL, Hiratzka LF, Hunt SA, Krumholz HM, Kushner FG, Lytle BW, Nishimura RA, Ornato JP, Page RL, Riegel B, Tarkington LG, Yancy CW, American College of Cardiology/American Heart Association Task Force on Practice G, American Association for Thoracic S and Society of Thoracic S (2008). ACC/AHA/HRS 2008 guidelines for device-based therapy of cardiac rhythm abnormalities: a report of the American College of Cardiology/American Heart Association Task Force on Practice Guidelines (Writing Committee to Revise the ACC/AHA/NASPE 2002 Guideline Update for Implantation of Cardiac Pacemakers and Antiarrhythmia Devices) developed in collaboration with the American Association for Thoracic Surgery and Society of Thoracic Surgeons. J Am Coll Cardiol.

[12] Lee DS, Krahn AD, Healey JS, Birnie D, Crystal E, Dorian P, Simpson CS, Khaykin Y, Cameron D, Janmohamed A, Yee R, Austin PC, Chen Z, Hardy J, Tu JV (2010). Evaluation of early complications related to de novo cardioverter defibrillator implantation: insights from the Ontario ICD database. J Am Coll Cardiol.

[13] Krahn AD, Lee DS, Birnie D, Healey JS, Crystal E, Dorian P, Simpson CS, Khaykin Y, Cameron D, Janmohamed A, Yee R, Austin PC, Chen Z, Hardy J, Tu JV (2011). Predictors of short-term complications after implantable cardioverter-defibrillator replacement/clinical perspective. Circ Arrhythm Electrophysiol.

[14] Vijgen J, Botto G, Camm J, Hoijer CJ, Jung W, Le Heuzey JY, Lubinski A, Norekval TM, Santomauro M, Schalij M, Schmid JP, Vardas P (2004). Consensus statement of the European Heart Rhythm Association: updated recommendations for driving by patients with implantable cardioverter defibrillators. [105 refs]. Eur J Cardiovasc Nurs.

[15] Johansson I, Stromberg A (2010). Experiences of driving and driving restrictions in recipients with an implantable cardioverter defibrillator—the patient perspective. J Cardiovasc Nurs.

[16] Shea JB (2004). Quality of life issues in patients with implantable cardioverter defibrillators: driving, occupation, and recreation. AACN Adv Crit Care.

[17] Wennberg JE. Unwarranted variations in healthcare delivery: implications for academic medical centres. Br Med J. 2002;325:961. 10.1136/bmj.325.7370.961 PMC1124450 12399352

[18] O’Connor AM, Stacey D, Entwistle V, Llewellyn-Thomas H, Rovner D, Holmes-Rovner M, Tait V, Tetroe J, Fiset V, Barry M, Jones J. Decision aids for people facing health treatment or screening decisions. Cochrane Database Syst Rev. 2003;(1):CD001431. doi:10.1002/14651858:CD001431. 10.1002/14651858.CD001431 12804407

[19] Stacey D, Bennett CL, Barry MJ, Col NF, Eden KB, Holmes-Rovner M, Llewellyn-Thomas H, Lyddiatt A, Legare F, Thomson R. Decision aids for people facing health treatment or screening decisions. [Update in Cochrane Database Syst Rev. 2014;(1):CD001431; PMID: 24470076], [Update of Cochrane Database Syst Rev. 2009;(3):CD001431; PMID: 19588325]. Cochrane Database Syst Rev. 2014:CD001431. 10.1002/14651858.CD001431.pub4 24470076

[20] Carroll SL, Strachan PH, de Laat S, Schwartz L, Arthur HM (2013). Patients’ decision making to accept or decline an implantable cardioverter defibrillator for primary prevention of sudden cardiac death. Health Expect.

[21] Strachan PH, Carroll SL, de Laat S, Schwartz L, Arthur HM (2011). Patients’ perspectives on end-of-life issues and implantable cardioverter defibrillators. J Palliat Care.

[22] Medical Research Council Health S, Public Health Research B (2000). A framework for development and evaluation of RCT’s for complex interventions to improve health.

[23] Carroll SL, McGillion M, Stacey D, Healey JS, Browne G, Arthur HM, Thabane L (2013). Development and feasibility testing of decision support for patients who are candidates for a prophylactic implantable defibrillator: a study protocol for a pilot randomized controlled trial. Trials.

[24] Elwyn G, O’Connor AM, Bennett C, Newcombe RG, Politi M, Durand MA, Drake E, Joseph-Williams N, Khangura S, Saarimaki A, Sivell S, Stiel M, Bernstein SJ, Col N, Coulter A, Eden K, Harter M, Rovner MH, Moumjid N, Stacey D, Thomson R, Whelan T, van der WT, Edwards A (2009). Assessing the quality of decision support technologies using the International Patient Decision Aid Standards instrument (IPDASi). PLoS ONE.

[25] Elwyn G, O’Connor A, Stacey D, Volk R, Edwards A, Coulter A, Thomson R, Barratt A, Barry M, Bernstein S, Butow P, Clarke A, Entwistle V, Feldman-Stewart D, Holmes-Rovner M, Llewellyn-Thomas H, Moumjid N, Mulley A, Ruland C, Sepucha K, Sykes A, Whelan T (2006). Developing a quality criteria framework for patient decision aids: online international Delphi consensus process. BMJ.

[26] O’Connor AM (2006). Ottawa Decision Support Framework.

[27] Carroll SL, McGillion M, Cheng J, Stacey D, Healey JS, Browne G, Arthur HM, Thabane L (2014). Adverse outcome probabilities of implantable defibrillators: application to a patient decision aid. Heart Rhythm J.

[28] Carroll SL, McGillion M, McGrath C, Stacey D, Healey J, Browne G, Thabane L, Arthur HM (2013). Application of a delphi method to develop a patient decision aid for implantable cardioverter defibrillator candidates. Circ Qual Care Outcomes Res.

[29] Carroll SL, McGillion M, Healey JS, Foster G, Browne G, Sum C, Stacey D, Thabane L. Engaging patients and families during development of a patient decision aid (PtDA) for an implantable defibrillator—acceptability results. Can J Cardiol. 2014;30(10):S365–S366.

[30] Bennett C, Graham ID, Kristjansson E, Kearing SA, Clay KF, O’Connor AM (2010). Validation of a Preparation for Decision Making Scale. Patient Educ Couns.

[31] Graham ID and O’Connor AM. User manual-preparation for Decision Making Scale. 2010:1–3. https://decisionaid.ohri.ca/docs/develop/User_Manuals/UM_PrepDM.pdf. 10.1016/j.pec.2009.05.012 19560303

[32] McHorney CA, War JE, Lu JFR, Sherbourne CD (1994). The MOS 36-Item Short-Form Health Survey (SF-36): III. Tests of data quality, scaling assumptions, and reliability across diverse patient groups. Med Care.

[33] McHorney CA, Ware JE, Raczek AE (1993). The MOS 36-Item Short-Form Health Survey (SF-36): II. Psychometric and clinical tests of validity in measuring physical and mental health constructs. Med Care.

[34] Radloff LS (1977). The CES-D Scale: a Self-Report Depression Scale for research in the general population. Appl Psychol Meas.

[35] Radloff LS (1991). The use of the Center for Epidemiologic Studies Depression Scale in adolescents and young adults. J Youth Adolesc.

[36] Miller WC, Anton HA, Townson AF (2008). Measurement properties of the CESD scale among individuals with spinal cord injury. Spinal Cord.

[37] Kuptniratsaikul V, Chulakadabba S, Ratanavijitrasil S (2002). An instrument for assessment of depression among spinal cord injury patients: comparison between the CES-D and TDI. J Med Assoc Thail.

[38] Failde I, Ramos I (2000). Validity and reliability of the SF-36 Health Survey Questionnaire in patients with coronary artery disease. J Clin Epidemiol.

[39] Jenkinson C, Wright L, Coulter A (1994). Criterion validity and reliability of the SF-36 in a population sample. Qual Life Res.

[40] O'Connor AM. User Manual - Decisional Conflict Scale (16 item question format) [document on the internet]. Ottawa: Ottawa Hospital Research Institute; Copyright 1993 [updated 2010; cited 2011 07 25]. 16p. Available from https://decisionaid.ohri.ca/docs/develop/User_Manuals/UM_Decisional_Conflict.pdf.

[41] O’Connor AM (1995). Validation of a decisional conflict scale. Med Decis Mak.

[42] O’Connor AM, Bennett CL, Stacey D, Barry M, Col NF, Eden KB, Entwistle VA, Fiset V, Holmes-Rovner M, Khangura S, Llewellyn-Thomas H, Rovner D. Decision aids for people facing health treatment or screening decisions. Cochrane Database Syst Rev. 2009;(3):CD001431. 10.1002/14651858.CD001431.pub2 19588325

[43] Legare F, Kearing S, Clay K, Gagnon S, D’Amours D, Rousseau M, O’Connor A (2010). Are you SURE?: Assessing patient decisional conflict with a 4-item screening test. Can Fam Physician.

[44] Ferron Parayre A, Labrecque M, Rousseau M, Turcotte S, Legare F (2014). Validation of SURE, a four-item clinical checklist for detecting decisional conflict in patients. Med Decis Mak.

[45] Teare M, Dimairo M, Shephard N, Hayman A, Whitehead A, Walters S (2014). Sample size requirements to estimate key design parameters from external pilot randomised controlled trials: a simulation study. Trials.

[46] Cocks K, Torgerson DJ. Sample size calculations for pilot randomized trials: a confidence interval approach. J Clin Epidemiol. 2012;66:197–201. 10.1016/j.jclinepi.2012.09.002 23195919

[47] Thabane L, Ma J, Chu R, Cheng J, Ismaila A, Rios L, Robson R, Thabane M, Giangregorio L, Goldsmith C (2010). A tutorial on pilot studies: the what, why and how. BMC Med Res Methodol.

[48] Calvert M, Blazeby J, Altman DG, Revicki DA, Moher D, Brundage MD, Group CP (2013). Reporting of patient-reported outcomes in randomized trials: the CONSORT PRO extension. JAMA.

[49] Morgan M, Deber R, Llewellyn-Thomas H, Gladstone P, Cusimano R, O’Rourke K, Tomlinson G, Detsky A (2000). Randomized, controlled trial of an interactive videodisc decision aid for patients with ischemic heart disease. J Gen Intern Med.

[50] Man-Son-Hing M, Laupacis A, O’Connor AM, Hart RG, Feldman G, Blackshear JL, Anderson DC (2000). Development of a decision aid for atrial fibrillation who are considering antithrombotic therapy. J Gen Intern Med.

[51] Thomson RG, Eccles MP, Steen IN, Greenaway J, Stobbart L, Murtagh MJ, May CR (2007). A patient decision aid to support shared decision-making on anti-thrombotic treatment of patients with atrial fibrillation: randomised controlled trial. Qual Saf Health Care.

[52] Laupacis A, O’Connor AM, Drake ER, Rubens FD, Robblee JA, Grant FC, Wells PS (2006). A decision aid for autologous pre-donation in cardiac surgery—a randomized trial. Patient Educ Couns.

[53] McCaffery KJ, Holmes-Rovner M, Smith SK, Rovner D, Nutbeam D, Clayman ML, Kelly-Blake K, Wolf MS, Sheridan SL (2013). Addressing health literacy in patient decision aids. BMC Med Inform Decis Mak.

[54] Carroll SL, Strachan P, Schwartz L, Arthur H (2004). Patients’ decisions about receiving an ICD for primary prevention of sudden cardiac death—information gathering in patient waiting rooms from “armchair expert” ICD veterans. Eur J Cardiovasc Nurs.

[55] Schwartz MD, Valdimarsdottir HB, DeMarco TA, Peshkin BN, Lawrence W, Rispoli J, Brown K, Isaacs C, O’Neill S, Shelby R, Grumet SC, McGovern MM, Garnett S, Bremer H, Leaman S, O’Mara K, Kelleher S, Komaridis K (2009). Randomized trial of a decision aid for BRCA1/BRCA2 mutation carriers: impact on measures of decision making and satisfaction. Health Psychol.

[56] Berry DL, Halpenny B, Hong F, Wolpin S, Lober WB, Russell KJ, Ellis WJ, Govindarajulu U, Bosco J, Davison BJ, Bennett G, Terris MK, Barsevick A, Lin DW, Yang CC, Swanson G (2013). The personal patient profile-prostate decision support for men with localized prostate cancer: a multi-center randomized trial. Urol Oncol.

[57] Carroll S, Pannag J, Hutchings S, Embuldeniya G, Mcgillion M, Strachan P, Stacey D (2016). Clarifying patients’ values for benefits and harms of initiating implantable defibrillators—easier said than done. Eur J Cardiovasc Nurs.

