# Supplementary material for: Evaluating the feasibility of conducting a trial using a patient decision aid in implantable cardioverter defibrillator candidates: a randomized controlled feasibility trial
Source: Pilot Feasibility Stud. 2017 Nov 21;3:49. doi: 10.1186/s40814-017-0189-9 (PMC5697082; doi:10.1186/s40814-017-0189-9)
Supplement: Supplementary file 1 — Knowledge and value items included in the Patient Decision Aid. (PDF 76 kb) [file 40814_2017_189_MOESM1_ESM.pdf]

Additional file 1- Knowledge and VALUE items

Knowledge questions included in Patient Decision Aid

1.	The likelihood that an ICD can correct a dangerous fast heart rhythm, should it occur, is very high.
2.	An ICD can alter heart failure or other heart symptoms including breathing difficulty, feeling tired, and/or chest pain.
3.	The battery in an ICD can last….. 2-3 years; 5-8 years; I am unsure
4.	If the ICD delivers therapy, I could face temporary driving restrictions.
5.	In the future, I can choose to ask my doctor to turn off (deactivate) ICD therapy.

Patients are asked to test what they know about the key facts by choosing, True, False, or unsure (with the exception of item 3).

Value items included in Patient Decision Aid

How important is it that ...	Not Important				Very Important
How important is it to you to lower your chances of a sudden cardiac death?	1	2	3	4	5
How important is it to you to have the peace of mind that a dangerous fast heart rhythm could be corrected?	1	2	3	4	5
How important is it to you to avoid complications from an ICD?	1	2	3	4	5
How important is it for you to avoid shocks from an ICD?	1	2	3	4	5
How important is it for you to die naturally when your time comes? (i.e. without life prolonging technology)	1	2	3	4	5
How important is it at this time in your life to choose medical treatments that could improve how you feel physically? (like breathing, feeling tired, walking, or climbing stairs)	1	2	3	4	5

Patients were asked to choose how much each of the items mattered to them by marking an "**X**" on the scale from 1-5.
